# Gene set enrichment analysis of microarray data from *Pimephales promelas *(Rafinesque), a non-mammalian model organism

**DOI:** 10.1186/1471-2164-12-66

**Published:** 2011-01-26

**Authors:** Michael A Thomas, Luobin Yang, Barbara J Carter, Rebecca D Klaper

**Affiliations:** 1 Department of Biological Sciences, Idaho State University, Stop 8007, 921 S 8thAve, Pocatello Idaho 83209-8007, USA; 2 EcoArray, Inc., Interstate Office Park, Suite 50, 4949 SW 41stBoulevard, Gainesville, FL 32608-5061, USA; 3 Great Lakes WATER Institute, School of Freshwater Sciences, University of Wisconsin - Milwaukee, 600 E. Greenfield Ave., Milwaukee, WI 53204-2944, USA

## Abstract

**Background:**

Methods for gene-class testing, such as Gene Set Enrichment Analysis (GSEA), incorporate biological knowledge into the analysis and interpretation of microarray data by comparing gene expression patterns to pathways, systems and emergent phenotypes. However, to use GSEA to its full capability with non-mammalian model organisms, a microarray platform must be annotated with human gene symbols. Doing so enables the ability to relate a model organism's gene expression, in response to a given treatment, to potential human health consequences of that treatment. We enhanced the annotation of a microarray platform from a non-mammalian model organism, and then used the GSEA approach in a reanalysis of a study examining the biological significance of acute and chronic methylmercury exposure on liver tissue of fathead minnow (*Pimephales promelas*). Using GSEA, we tested the hypothesis that fathead livers, in response to methylmercury exposure, would exhibit gene expression patterns similar to diseased human livers.

**Results:**

We describe an enhanced annotation of the fathead minnow microarray platform with human gene symbols. This resource is now compatible with the GSEA approach for gene-class testing. We confirmed that GSEA, using this enhanced microarray platform, is able to recover results consistent with a previous analysis of fathead minnow exposure to methylmercury using standard analytical approaches. Using GSEA to compare fathead gene expression profiles to human phenotypes, we also found that fathead methylmercury-treated livers exhibited expression profiles that are homologous to human systems & pathways and results in damage that is similar to those of human liver damage associated with hepatocellular carcinoma and hepatitis B.

**Conclusions:**

This study describes a powerful resource for enabling the use of non-mammalian model organisms in the study of human health significance. Results of microarray gene expression studies involving fathead minnow, typically used for aquatic ecological toxicology studies, can now be used to generate hypotheses regarding consequences of contaminants and other stressors on humans. The same approach can be used with other model organisms with microarray platforms annotated in a similar manner.

## Background

One of the challenges facing researchers conducting microarray studies is deriving meaning from lists of thousands of differentially expressed genes among the phenotypes examined [1-3]. A relatively new approach for systems-based analyses involves testing for enrichment of gene classes or sets; the most popular method employing gene-class analysis is Gene Set Enrichment Analysis (GSEA [4]). GSEA tests whether a set of genes, defined *a priori*, is enriched in expression in one treatment relative to another. Each GSEA set consists of genes united by a shared association (e.g., functional classification, pathway or disease state), leveraging prior knowledge into the analysis and thereby providing an advantage over an approach in which only individual genes are examined. An enrichment score is calculated for each set to reflect the distribution of set constituents across a list of genes ranked by correlation with the experimental treatment. A higher enrichment score corresponds to a shifting of gene set constituents towards either end of the ranked list representing strongly positive or negative correlations. For a specific microarray experiment, GSEA tests whether genes from a given set are randomly distributed or, alternatively, are up- or down-regulated in one phenotype relative to the other.

The statistical significance of a set's enrichment score is determined by comparison to a distribution of scores generated by permuting the ranked list by phenotype class. A measure of expected false discovery rate (FDR) is used to refine the significance of high-scoring sets when a large collection of gene sets is analyzed.

The GSEA approach uses a modified Kolmogorov-Smirnov test. Some authors have criticized the GSEA approach for being a Rube Goldberg Machine-like solution to a problem that could be adequately solved with a simpler instrument, such as a *X *^2 ^test that assumes a normal distribution of scores [5]. While it is unclear which statistical approach best tests for gene set enrichment, it is generally accepted that the GSEA approach is able to provide novel insights from complex expression patterns [2,3].

GSEA is human-centric: the GSEA platform [6] is built around human genome data and HUGO (Human Genome Organization) gene symbols, a standard vocabulary of gene terms [7]; array elements without associated HUGO symbols are ignored by GSEA. For each known human gene, the HUGO Gene Nomenclature Committee (HGNC) approves a single gene name and symbol. Researchers not using human microarray platforms must annotate elements in their array with HUGO terms in order to use GSEA along with its database of gene sets. This is clearly a non-trivial task for distantly related model organisms, as a substantial proportion of genetic elements on such an array will have no known human homolog. However, this enhanced annotation allows access to thousands of curated gene sets available in the Molecular Signatures Database (MSigDB [4,8]) that leverage human health knowledge, allow meaningful comparisons between humans and distantly related model organisms, and potentially provide novel insights into human health.

McGary et al. (2010) demonstrated the value of comparisons between humans and distantly related model organisms for understanding the evolution of emergent phenotypes arising from sets of conserved genes [9]. In that study, sets of genes associated with specific human genetic disorders were mapped to sets of homologous genes in model organisms associated with functions distinct from the human genes (e.g., human X-linked breast cancer vs. a high frequency of male progeny in *C. elegans*). That approach was used to identify novel candidate genes for the human disorder.

Similarly, a GSEA-based analysis could leverage phenotype homology with two different approaches. First, starting with a gene set associated with a given human disorder (e.g., genes associated with a given human autoimmune disorder characterized by an unknown environmental trigger), GSEA could be used to assay a number of conditions in which those genes might be differentially expressed in an appropriate model organism. In this way, GSEA could provide novel insights into that disorder by generating hypotheses about circumstances under which the human disorder is mimicked, exacerbated or even triggered. Second, GSEA could be used to predict the human health consequences of a given treatment or condition (e.g., selenium contamination and concentration in streams and wetlands, as in [10]) by comparing the gene expression profile associated with that contaminant (in an appropriate model organism) to a collection of candidate human gene sets chosen to represent a range of reasonable pathways, functions or phenotypes of interest.

Here, we describe an enhanced annotation of the EcoArray fathead minnow 15 k microarray (EcoArray, Gainesville, Florida) using HUGO symbols [11]. This enhanced microarray resource allows analyses using the GSEA approach and comparisons between fathead expression and sets associated with human health.

To test the ability to GSEA to recover results consistent with standard microarray analyses, we reanalyzed a previous study of fathead minnows, *Pimephales promelas *(Rafinesque), that used the EcoArray fathead minnow 15 k gene microarray platform to examine gene expression changes in response to methylmercury exposure [12,13]. That study considered genes with greater than two-fold differences from control in liver tissue from fish exposed to methylmercury over short (96 hour, "acute") or long (600 day, "chronic") exposure periods. They identified 650 genes that exceeded this threshold following acute treatment and 267 genes following chronic treatment. Examining these genes with the FatiGO functional profiling tool [1,14], they identified Gene Ontology (GO) categories [15] found to be enriched in response to the acute and chronic methylmercury treatments. In all treatments, analyses identified methylmercury-induced changes in expression of apoptosis-associated genes, including caspase, tumor necrosis factor and fatty acid synthase.

In order to test the ability of GSEA to conduct meaningful comparisons of human disease-associated sets with model organism expression profiles, we compared liver gene expression profiles from the fathead minnow methylmercury treatments [12,13] to MSigDB-derived sets associated with gene expression in human livers damaged by hepatocellular carcinoma (HCC) and hepatitis B. We predicted that these sets would be enriched in the fish expression profiles, reflecting similar processes associated with generalized liver damage. These comparisons were designed to investigate the broader biological significance of methylmercury exposure by fathead minnows while providing a clear comparison of liver damage in a non-mammalian model organism to human liver damage associated with specific disorders.

## Results

### Annotation of EcoArray 15 k fathead minnow microarray for GSEA analysis

We identified 12,032 HUGO symbols for the 15,208 elements on the EcoArray fathead minnow microarray platform (79%). The annotated fish genes include homology to 10,069 unique HUGO symbols, about 36% of the ~28,000 symbols in the HUGO database. This number of human homologs is consistent with the number identified (10-11,000) for five other fish species using similar methods [16], given that the 15,208 elements on the fathead array were selected in part for their degree of conservation with other species [12,13].

Gene duplication in the fish lineage may have led to cases where more than one fish array elements mapped to the same human HUGO symbol. There were 1679 array elements that shared a HUGO symbol association with at least one other array element. These include situations where 1) fathead genes are represented by more than one array element; and 2) two (or more) fathead genes were homologous to the same human gene due to gene duplication in the fish lineage. Using the EST sequence data, it is difficult to distinguish between these two possibilities; GSEA assumes that all such duplicates fall into the first category. We concluded that cases from the second category would not unduly influence the interpretation of the analyses since the post-duplication sister genes likely have similar functions.

Similarly, gene duplication in the mammalian lineage has led to cases in which a single fish array element mapped equally to more than one human protein (and HUGO symbol). Of the ~250 such cases, in which the BLAST search identified two or more hits with equal e-values (generally 0.0), we selected the hit with the higher score (see Materials & Methods). These cases were examined individually.

### GSEA analysis results for GO categories derived from Klaper et al. study

Of the 26 GO-BP (Biological Processes) classes identified by the Klaper et al. [12,13] study (using FatiGO), 14 sets for acute and 12 sets for chronic treatments, GSEA identified six significantly enriched sets in the acute treatment (Table 1) and no sets in the chronic treatment (Table 2). Consistent with the Klaper study, GSEA identified the majority of sets to have experienced up-regulation (irrespective of statistical significance of the enrichment) in the treatments relative to the controls. Nearly all the enriched gene sets were associated with metabolic and biosynthetic processes.

**Table 1:** GSEA tests for GO classes enriched following acute treatment

GO term	GO definition	Size	ES	NES	NOM p-value
**UP-regulated in treatment**:					
GO:0042254	**Ribosome biogenesis and assembly**	14	0.708	1.591	0.000
GO:0006364	**rRNA processing**	11	0.717	1.510	0.000
GO:0015031	**Protein transport**	122	0.437	1.614	0.014
GO:0006754	**ATP biosynthesis**	56	0.438	1.360	0.014
GO:0006486	Protein amino acid glycosylation	75	0.378	1.280	0.133
GO:0006605	**Protein targeting**	85	0.436	1.557	0.013
GO:0009058	Biosynthesis	327	0.324	1.315	0.149
GO:0045045	Secretory pathway	59	0.422	1.448	0.064
GO:0007046	Ribosome biogenesis	94	0.552	1.553	0.054
GO:0006457	Protein folding	44	0.466	1.387	0.086
GO:0006633	Fatty acid biosynthesis	11	0.411	1.162	0.296
GO:0006888	ER to golgi vesicle mediated transport	11	0.365	0.864	0.676
**DOWN-regulated in treatment**:					
GO:0009165	Nucleotide biosynthesis	12	-0.379	-1.047	0.458
**GO:0007498**	**Mesoderm development**	10	-0.647	-1.610	0.000

Results for gene sets representing each GO-BP class identified by Klaper et al. [12,13], tested for enrichment by GSEA following acute methylmercury treatment. GO terms and definitions can be used to retrieve each set (and constituent genes) from MSigDB [8]; size refers to the number of genes in the set; ES and NES are the enrichment scores and normalized enrichment scores (respectively) for the set; NOM p-value is the nominal p-value for the NES. Gene sets representing GO-BP classes with statistically significant normalized enrichment scores are highlighted in bold.

**Table 2:** GSEA tests for GO classes enriched following chronic treatment

GO term	GO definition	Size	ES	NES	NOM p-value
**UP-regulated in treatment**:					

GO:0006605	Protein targeting	85	0.387	1.416	0.054

GO:0016485	Protein processing	32	0.413	1.267	0.086

GO:0006897	Endocytosis	162	0.253	1.091	0.278

GO:0000226	Microtubule cytoskeleton organization and biogenesis	28	0.376	1.004	0.484

GO:0007409	Axonogenesis	29	0.362	0.992	0.614

GO:0045893	Positive regulation of transcription, DNA dependent	270	0.200	0.898	0.502

GO:0006887	Exocytosis	15	0.402	0.856	0.763

GO:0006289	Nucleotide-excision repair	14	0.287	0.842	0.806

GO:0007269	Neurotransmitter secretion	10	0.302	0.702	0.786

**DOWN-regulated in treatment**:					

GO:0006917	Induction of apoptosis	208	-0.286	-1.093	0.396

GO:0009615	Response to virus	21	-0.301	-1.198	0.130

GO:0001756	Somitogenesis	17	-0.356	-1.020	0.502

Results for each GO-BP class identified by Klaper et al. [12,13], tested for enrichment by GSEA following chronic methylmercury treatment. Size refers to the number of genes in the set; ES and NES are the enrichment scores and normalized enrichment scores (respectively) for the set; NOM p-value is the nominal p-value for the NES.

### GSEA analysis of the MSigDB C2 collection of gene sets

Due to the highly conservative nature of the false discover rate (FDR) implemented by GSEA, many gene sets from the MSigDB C2 collection ("curated gene sets from online pathway databases, publications in PubMed, and knowledge of domain experts" [4]) with statistically significant enrichment scores failed to surpass the FDR threshold and, consequently, could not be definitively identified as enriched following methylmercury treatment. Table 3 lists gene sets with normalized enrichment scores (NES, see Table captions for details) that were below a normalized p-value of 0.05 and a FDR of 0.25.

**Table 3:** GSEA tests for enrichment of MSigDB C2 gene sets

		Gene Set Name	Size	ES	NES	NOM p-value	FDR q-value
Acute exposure	Down-regulated	None					
							
	Up-regulated	XU_CBP_UP	19	0.633	1.987	0.000	0.051
		CALRES_MOUSE_DN	28	0.575	1.920	0.000	0.108
		BYSTRYKH_HSC_CIS_GLOCUS	82	0.516	1.889	0.000	0.097
		BYSTRYKH_HSC_BRAIN_CIS_GLOCUS	43	0.600	1.855	0.000	0.102
		PENG_GLUTAMINE_DN	209	0.519	1.828	0.000	0.130
		UVC_LOW_ALL_DN	38	0.473	1.817	0.000	0.121
		CMV_ALL_UP	64	0.530	1.764	0.000	0.214
		SANSOM_APC_LOSS5_UP	55	0.544	1.734	0.037	0.274
		BLEO_MOUSE_LYMPH_HIGH_24HRS_DN	31	0.669	1.730	0.000	0.255
		SHEPARD_CRASH_AND_BURN_MUT_VS_WT_UP	124	0.452	1.710	0.000	0.289
		PENG_RAPAMYCIN_DN	163	0.516	1.706	0.023	0.275
		PRMT5_KD_DN	19	0.572	1.694	0.038	0.298
		HSA00970_AMINOACYL_TRNA_BIOSYNTHESIS	29	0.702	1.690	0.029	0.286
		MRNA_SPLICING	44	0.633	1.689	0.000	0.266
		WANG_MLL_CBP_VS_GMP_DN	30	0.579	1.688	0.000	0.254
		JAIN_NEMO_DIFF	59	0.458	1.688	0.000	0.240
		HSA00563_GLYCOSYLPHOSPHATIDYLINOSITOL_ANCHOR_BIOSYNTHESIS	16	0.616	1.684	0.000	0.239
		BLEO_MOUSE_LYMPH_LOW_24HRS_DN	22	0.697	1.681	0.000	0.238
		OLDONLY_FIBRO_UP	28	0.485	1.674	0.000	0.243
		CMV_24HRS_UP	54	0.524	1.673	0.000	0.232
		KUROKAWA_5FU_IFN_SENSITIVE_VS_RESISTANT_DN	22	0.624	1.669	0.000	0.232
		CHANG_SERUM_RESPONSE_UP	109	0.480	1.668	0.000	0.224
		ROS_MOUSE_AORTA_UP	21	0.731	1.656	0.036	0.237
		UVB_NHEK2_DN	64	0.456	1.649	0.000	0.242
		SCHUMACHER_MYC_UP	44	0.504	1.649	0.023	0.234
		STEMCELL_COMMON_UP	149	0.458	1.643	0.023	0.242
		MYC_ONCOGENIC_SIGNATURE	137	0.406	1.641	0.000	0.239
		ZHAN_MMPC_SIM_BC_AND_MM	36	0.472	1.635	0.000	0.242
		AMINOACYL_TRNA_BIOSYNTHESIS	21	0.732	1.633	0.048	0.238
							
Chronic exposure	Down-regulated	TNFR1PATHWAY	24	-0.724	-1.968	0.000	0.026
		CASPASEPATHWAY	16	-0.684	-1.865	0.000	0.049
		DEATHPATHWAY	25	-0.719	-1.836	0.000	0.063
		HIVNEFPATHWAY	44	-0.593	-1.765	0.000	0.104
		MITOCHONDRIAPATHWAY	17	-0.785	-1.728	0.000	0.151
		TSA_PANC50_UP	25	-0.584	-1.684	0.000	0.216
							
	Up-regulated	None					

Results for MSigDB C2 gene sets (drawn from 1892 sets representing all systems, pathways and functions in that database), listing sets with NES scores that are both statistically significant and below the FDR threshold. Size refers to the number of genes in the set; ES and NES are the enrichment scores and normalized enrichment scores (respectively) for the set; NOM p-value is the nominal p-value for the NES, FDR q-vlaue is the false discovery rate ratio (values lower than 0.25 are considered to be passing as per Subramanian et al. [4]). One of these sets (in bold) is a liver associated set highlighted in Table 4.

In the acute treatment, GSEA identified 20 significantly enriched up-regulated sets under the FDR threshold (Table 3). These consisted of metabolic, biosynthetic and several cancer-associated sets. There were no significantly enriched down-regulated sets passing the FDR test; failing FDR (not listed in Table 3) were significantly down-regulated sets associated with cytotoxicity, which is of biological significance for hepatotoxicity and, potentially, for HCC.

In the chronic treatment, GSEA identified 6 significantly enriched down-regulated sets under the FDR threshold (Table 3). These primarily include apoptosis and caspase sets. There were no significantly enriched up-regulated sets passing the FDR test; failing FDR (not listed in Table 3) were significantly up-regulated metabolic and biosynthetic processes sets, which are of biological significance for hepatotoxicity and, potentially, for HCC.

### GSEA analysis results for human HCC and hepatitis B gene sets

A sub-set of the MSigDB C2 collection, consisting of 38 human gene sets associated with HCC and hepatitis B, was also tested against the *P. promelas *methylmercury treatments (see Materials & Methods and Table 4). In the acute treatment, GSEA found one down-regulated ("Gene up-regulated both by expression of Hepatitis B virus HBx protein in normal hepatoctyes and by HBV infection in liver samples with chronic active hepatitis") and two up-regulated sets ("Genes highly expressed in HCC resistant to 5-Fluorouracil + interferon" and "Genes highly expressed in interferon-resistant hepatoma cell lines vs. sensitive cell lines"). In the chronic treatment, GSEA found no down-regulated and one up-regulated set ("Genes highly expressed in hepatocellular carcinoma with poor survival").

**Table 4:** GSEA tests for enrichment of HCC- and hepatitis-associated gene sets

Gene Set Name	Size	ES	NES	NOM p-value
**Up-regulated in acute liver treatment**				

KUROKAWA_5FU_IFN_SENSITIVE_VS_RESISTANT_DN	25	0.585	1.642	0.025

WONG_IFNA_HCC_RESISTANT_VS_SENSITIVE_UP	10	0.507	1.407	0.046

**Down-regulated in acute liver treatment**				

HBX_HEP_UP	12	-0.540	-1.460	0.038

**Up-regulated in chronic liver treatment**				

HCC_SURVIVAL_GOOD_VS_POOR_DN	112	0.452	1.549	0.000

**Down-regulated in chronic liver treatment**				

None				

Results for gene sets representing Hepatocellular carcinoma (HCC) and hepatitis, tested for enrichment by GSEA following methylmercury treatment. Here, we test whether liver-associated sets are enriched following methylmercury exposure. Size refers to the number of genes in the set; ES and NES are the enrichment scores and normalized enrichment scores (respectively) for the set; NOM p-value is the nominal p-value for the NES. Descriptions of each set (and lists of constituent genes) can be found by searching for set names at the MSigDB [8].

## Discussion

### Comparisons of GSEA and FatiGO profiles

Our GSEA results are consistent with the FatiGO results of Klaper et al. [12,13]. While few of the FatiGO sets were significantly enriched in the GSEA analysis, all sets were up- or down-regulated in the same direction (mainly up-regulation) and the sets significant in the GSEA analysis tended to include those GO processes highlighted by Klaper et al. as being important consequences of liver methylmercury exposure. This includes predominantly up-regulation of metabolic and biosynthetic processes (following acute exposure) and down-regulation of apoptosis (following chronic exposure).

It is noteworthy that the GSEA-based approach uses the entirety of the ~12,000 annotated elements on the array, rather than only the most highly differentially expressed genes. This difference in the scope of analysis provides an explanation for why all FatiGO process terms were not significantly enriched in the GSEA analysis (and why some GSEA-enriched sets were not identified by FatiGO; see below). In other words, GSEA enrichment of a set containing many genes associated with a given function is somewhat different than a FatiGO-style analysis that reports the function of a single given up-regulated gene: While FatiGO asks whether any system or pathway is statistically over-represented in the (100 or so) most up-regulated genes (out of ~15,000 on the chip), GSEA asks if the dozens of genes associated with a given system or pathway are significantly collectively enriched (i.e. up- or down-regulated) in a study that simultaneously considers all annotated genes on a chip (~12,000 in our experiment).

There are important distinctions between the expression profiles associated with acute and chronic exposure, potentially reflecting metabolic changes occurring after prolonged exposure to a toxin (rather than short term, acute exposure) observed by other researchers [17]. This difference may be due to either accumulation of methylmercury over time or acclimation of the tissue over time. These differences also reflect the very different nature of the two experiments, performed by different labs using different protocols.

Overall, the C2 results were limited by a FDR due to the size of the MSigDB-C2 database. The few gene sets that passed this threshold were consistent with FatiGO results and the general conclusions of Klaper et al. [12,13]. Primarily, these include sets associate with up-regulation of metabolic and biosynthetic processes and down-regulation of caspase and apoptosis pathways. Published studies of apoptosis induction by methylmercury involved neurological [18] and reproductive systems [19], so it is not necessarily unexpected to observe the opposite response in the liver, the primary site of detoxification. The up-regulation of general metabolic processes might be involved in detoxification, although none of the sets we identified were specifically associated with DNA repair, which has been noted in similar experiments [17,20].

### Comparisons to human liver disease

The smaller, select group of gene sets, involving liver damage associated with HCC and hepatitis B, provided meaningful comparison between human liver disease and fish methylmercury exposure rather than simply a list of the altered biological functions (Table 4). The scores of these sets are similar to the scores of the highest-scoring sets from the broader analysis (those gene sets in Table 3).

Fathead minnow liver tissues following acute methylmercury treatment were enriched for a set (KUROKAWA [21]) composed of genes that differentiate among patients with advanced HCC with respect to response to chemotherapy treatment. Fish in the acute treatment are also enriched for genes highly expressed in interferon-resistant hepatoma cell lines (WONG [22]). This indicates a shared response between acute methylmercury exposure (in fish) and hepatocellular carcinoma (in humans), which is potentially useful for guiding the understanding of methylmercury toxicology.

Fathead minnow liver tissues following chronic methylmercury treatment were enriched for sets associated with HCC (HCC_survival, reviewed in [23]) and hepatitis B (Lizuka [24], HBX_NL [25]); see Table 4. These sets are indicative of a shared response between chronic exposure (in fish) and serious liver impairment (in humans) due to cancer and hepatitis infection. That both HCC and hepatitis B are enriched is not surprising, since there is a relationship between the two conditions [26].

While it is well known that other heavy metals are directly associated with cancer [27], for mercury it is less clear, despite indirect evidence involving liver cancer [28]. HCC is generally secondary to hepatitis infection or cirrhosis-associated liver damage by hepatotoxicity (caused by alcohol or non-alcohol toxins). Mercury induces oxidative stress, which leads to the enhanced biosynthesis of liver enzymes associated with antioxidant and toxic response systems [29] and genotoxicity [30]. Liver damage can occur from even topical exposure [31]. Using exhaustive histological and microarray comparisons between zebrafish livers and human cells exposed to mercury, Ung et al. [32] identified mercury-induced hepatotoxicity involving many of the same systems and pathways revealed by our GSEA-based study, including apoptosis, proteasomes, and other systems associated with toxicity response (in general) and hepatotoxicity (in particular).

## Conclusions

The comparison with Klaper et al. [12,13] is an indication that GSEA (and gene-class approaches, in general) are useful for model organisms that are not yet endowed with completely sequenced and fully annotated genomes. This provides both a systems-based gene-class analytical tool (generally only used with human, mouse and rat data, and, recently, zebrafish [33]) and a mechanism to compare to gene expression profiles from non-human model organisms to profiles associated with human phenotypes and another tool for understanding human health implications when using model organisms and a framework from which testable hypotheses can be generated. For these applications, the strength of gene-class approaches is in the *a priori *designation of sets to be tested. Also, in considering all genes on a chip rather than only those most up-regulated, gene-class analyses can identify subtle patterns easily missed by FatiGO-like analyses.

## Methods

### Fish and microarray work

Klaper et al. [12,13] describe the fish methylmercury exposure and microarray experimental design. The data for these experiments have been deposited at NCBI GEO [34] (accession GEO:GSE22261).

Briefly, fish were treated with either an acute or chronic exposure to methymercury. For the acute treatment, male adult fathead minnows were injected with a sublethal methylmercury concentration of 2.0 μg per g of body mass. There were 12 treated and 12 control fish (controls were given a vehicle injection without methylmercury). At the end of the 96-hour treatment period, livers were removed from euthanized fish (gonads were also collected but those data were not considered in the present GSEA reanalysis). For the chronic treatment, 90-day-old fathead minnows were exposed to methylmercury concentrations of 4.0 μg per g of dry mass (for treated fish) added to food, fed daily to the fish in quantities of approximately 5% of their body mass. (The fish food also contained naturally occurring methylmercury of ~0.05 μg per g of dry mass.) At the end of the 600 day treatment period, livers were removed from euthanized fish (as in the acute treatment, gonads were also collected).

The microarray data were normalized using Gene Spring version GX10.0.1. A standard "thresholding" substitution was conducted, in which expression values below 0.01 were set to 0.01. This was performed to remove very small or negative expression values prior to log-transforming the data, in order to eliminate large negative or missing values in the normalized data. The baseline was set to the median of all data. There was a percentile shift to the 50th percentile, and each spot was normalized to the median of all spots.

### Annotation of EcoArray 15 k fathead minnow microarray for GSEA analysis

The EcoArray Fathead Minnow 8 × 15K Microarray v1.0 platform (NCBI GEO accession GEO:GPL7351) was enhanced with human HUGO gene symbols. To accomplish this, we conducted BLASTX searches using 6-frame translated full-length EST sequences (representing each array element) against the human RefSeq protein database (November 2009 release). We used the BLOSUM62 matrix and default protein search options, after determining that these were the correct settings given the sequence divergence between fish and mammals. The e-value threshold was set to 0.01 and the 10 best matching sequences were reported. This relatively high e-value allowed us to see the range of hits recovered by BLAST; for purposes of selecting a BLAST hit for our annotation, only hits with e-values smaller than 10^-10 ^were considered.

We developed custom Perl scripts to parse BLAST output files and extract the RefSeq protein ID from the hit with the lowest e-value. We found 10575 elements with hits having an e-value lower than < 10^-10^. When there were multiple hits with equally low e-values, all were extracted and converted into HUGO symbols and retained for inspection. Generally, when more than one protein was found by BLAST, they shared a single HUGO symbol.

When the BLAST of a given EST sequence search had highly significant hits on multiple RefSeq protein IDs, we found they were generally isoforms of the same gene. In other cases, the results were individually curated to determine which gene symbol to use, if any. Generally, when the e-value was the same (e.g., 0.0), we chose the symbol associated with the highest score.

To convert a RefSeq protein ID into a HUGO value, we used the Babelomics ID converter tool [35]. We confirmed this symbol by obtaining the NCBI-derived HUGO symbol, along with its known aliases, for that protein ID and comparing it to the Babelomics-derived symbol. We further compared these two symbols to the HGNC-derived HUGO symbol, which had to be mapped through the RefSeq mRNA ID: first, we extracted the RefSeq mRNA ID from NCBI for each RefSeq protein ID; we then downloaded the database of all gene symbols, their aliases, and RefSeq mRNA IDs from the HGNC website. For each protein ID, via the mRNA ID, we were able to identify the appropriate HGNC HUGO symbol and compare it to the Babelomics- and NCBI-derived symbols. These steps ensured that we assigned an appropriate HUGO symbol to each RefSeq protein ID, and that this was the primary symbol, rather than an alias.

In order to support the result of the fathead EST to human RefSeq protein search, we also conducted a BLASTX search of each fathead ESTs against the zebrafish RefSeq protein database (June 21, 2010 version). We extracted the zebrafish RefSeq protein IDs if there is a significant BLAST hit with a low e-value (<10^-10^). We then downloaded the zebrafish to human orthology data file from ZFIN (http://zfin.org) and compared the zebrafish symbol to the human HUGO gene symbols; when these did not agree, they were human-curated (differences generally involved aliases).

We assembled this enhanced annotation in a GSEA-formatted file, EcoArray_Fathead.chip (additional file 1). Finally, for each gene element in this file, we crosschecked the symbol we assigned with those in GENE_SYMBOL.chip (from GSEA) to ensure that our symbol was the GSEA-recognized HUGO symbol, rather than an alias.

### Conducting GSEA analyses

Data files were created to GSEA specifications for the HUGO-annotated microarray (.CHIP format), normalized expression values from Klaper et al. [12,13] (.GCT format) paired with phenotype descriptions (.CLS format), and all gene sets used in GSEA analyses (.GMX format). These files are available as additional files 2, 3, 4, 5, 6.

Analyses used GSEA release 2.06 and MSigDB release 2.5. Weighted enrichment scores were calculated using gene expression lists ranked by signal-to-noise ratio. The maximum gene set size was set to 500 genes; the minimum gene set size was set to 10 genes; the number of permutations was set to 1000. For details of GSEA parameter usage, see Subramanian et al. [4] and the GSEA web site [6].

Gene sets were examined to ensure they contained only GSEA-recognized primary HUGO symbols, rather than aliases or unapproved symbols. This was accomplished through the use of a custom PHP script that compared each gene in a given set to the GENE_SYMBOLS.chip file (see above) containing a list of HUGO symbols with accepted aliases. Gene set components listed as aliases in this file were replaced with the appropriate HUGO symbol.

Three groups of analyses were conducted; each group consisted of a number of gene sets, with each set tested against one or both of the acute & chronic treatments relative to their respective control.

In the first group of analyses, we tested gene sets corresponding to GO-BP classes identified by the Klaper FatiGO analysis (Appendix II) identified as enriched by acute or chronic treatments. We eliminated gene sets that contained insufficient or excessive numbers of genes for GSEA analysis (leaving 14 and 12 sets for acute and chronic treatments, respectively). For each methylmercury treatment tested by GSEA, only sets corresponding to GO categories found by Klaper et al. *for that treatment *were considered in GSEA tests. These analyses were designed to test whether GSEA results were consistent with conclusions drawn by Klaper et al. [12,13].

Second, we tested all sets from the MSigDB collection containing canonical pathways and chemical and genetic perturbations (MSigDB C2, containing 1892 sets, of which 1617 were specific to human) against acute and chronic liver treatments.

Third, a more limited subset of the C2 collection, consisting of curated human gene sets associated with normal liver function, hepatocellular carcinoma (HCC) and hepatitis infection were tested against the fathead minnow liver treatments. These analyses were designed to highlight expression profiles in response to exposure to methylmercury that are similar to profiles associated with other liver insults in humans. These sets were obtained by searching the MSigDB C2 database for human gene sets using the search terms "liver OR hepatocellular OR hepatitis" in which 38 gene sets were recovered. These sets were pruned to 24 sets, eliminating sets with fewer than 10 genes with homologs in the EcoArray annotated microarray.

Gene sets from each of the three collections were tested for enrichment among the ~12,000 annotated genes from the EcoArray 15 k chip, ranked by signal-to-noise ratio. The nominal p-value reports the significance for a given set in an analysis of a collection of sets in which the researcher is specifically interested (like our 24 human liver sets). However, in an exploratory analysis (like the 1892 C2 collection of functional sets), an FDR test is critical to control for multiple comparisons, as it accounts for potential overlap in the gene sets (i.e., a given gene may appear in several sets).

## Authors' contributions

RDK conceived the original study from which data for this study were derived. MAT and RDK conceived this re-analysis. LY annotated the EcoArray fathead minnow microarray for GSEA analysis. BJC processed the original microarrays and conducted the Genespring analyses. MAT performed the new GSEA analyses, integrated these with the original GO analyses, and wrote the manuscript with RDK. All authors read and approved the final manuscript.

## Supplementary Material

Additional file 1 **EcoArrayFathead15 k.chip**. The list of fathead minnow elements from the EcoArray 15 k chip, annotated in this study and formatted for GSEA analysis. Click here for file

Additional file 2 **Liver-Acute.GMX**. The list of genes used in the acute liver analysis, formatted for GSEA. Click here for file

Additional file 3 **Liver-Chronic.GMX**. The list of genes used in the chronic liver analysis, formatted for GSEA. Click here for file

Additional file 4 **Liver-HCC-hepatitis-human2.GMX**. The list of genes used in the human liver analysis, formatted for GSEA. Click here for file

Additional file 5 **MeHg-data.GCT**. The raw data from the methylmercury experiment, formatted for GSEA. Click here for file

Additional file 6 **MeHg-phenotypes.CLS**. The phenotypes in the methylmercury experiment, formatted for GSEA. Click here for file
